# Inhibition of endocytic uptake of severe acute respiratory syndrome coronavirus 2 and endo-lysosomal acidification by diphenoxylate

**DOI:** 10.1128/aac.00341-24

**Published:** 2024-05-14

**Authors:** Jin Soo Shin, Yejin Jang, Dong-Su Kim, Eunhye Jung, Myoung Kyu Lee, Byungil Kim, Sunjoo Ahn, Yeonju Shin, Su San Jang, Chang Soo Yun, Jongman Yoo, Young Chang Lim, Soo Bong Han, Meehyein Kim

**Affiliations:** 1 Infectious Diseases Therapeutic Research Center, Korea Research Institute of Chemical Technology (KRICT), Daejeon, Republic of Korea; 2 Therapeutics and Biotechnology Division, Korea Research Institute of Chemical Technology (KRICT), Daejeon, Republic of Korea; 3 CHA Organoid Research Center, CHA University, Seongnam, Gyeonggi-do, Republic of Korea; 4 Department of Otorhinolaryngology-Head and Neck Surgery, The Research Institute, Konkuk University School of Medicine, Seoul, Republic of Korea; 5 Medicinal Chemistry and Pharmacology, University of Science and Technology (UST), Daejeon, Republic of Korea; IrsiCaixa Institut de Recerca de la Sida, Barcelona, Spain

**Keywords:** SARS-CoV-2, antiviral, diphenoxylate, endocytosis, endo-lysosomal acidification

## Abstract

Cell culture-based screening of a chemical library identified diphenoxylate as an antiviral agent against severe acute respiratory syndrome coronavirus 2 (SARS-CoV-2). The observed 50% effective concentrations ranged between 1.4 and 4.9 µM against the original wild-type strain and its variants. Time-of-addition experiments indicated that diphenoxylate is an entry blocker targeting a host factor involved in viral infection. Fluorescence microscopic analysis visualized that diphenoxylate prevented SARS-CoV-2 particles from penetrating the cell membrane and also impaired endo-lysosomal acidification. Diphenoxylate exhibited a synergistic inhibitory effect on SARS-CoV-2 infection in human lung epithelial Calu-3 cells when combined with a transmembrane serine protease 2 (TMPRSS2) inhibitor, nafamostat. This synergy suggested that efficient antiviral activity is achieved by blocking both TMPRSS2-mediated early and endosome-mediated late SARS-CoV-2 entry pathways. The antiviral efficacy of diphenoxylate against SARS-CoV-2 was reproducible in a human tonsil organoids system. In a transgenic mouse model expressing the obligate SARS-CoV-2 receptor, human angiotensin-converting enzyme 2, intranasal administration of diphenoxylate (10 mg/kg/day) significantly reduced the viral RNA copy number in the lungs by 70% on day 3. This study underscores that diphenoxylate represents a promising core scaffold, warranting further exploration for chemical modifications aimed at developing a new class of clinically effective antiviral drugs against SARS-CoV-2.

## INTRODUCTION

After severe acute respiratory syndrome coronavirus 2 (SARS-CoV-2) was first identified in an endemic area of China in December 2019, it spread rapidly worldwide and caused the COVID-19 pandemic (1). Its high human-to-human transmissibility and mortality resulted in a huge number of confirmed infection cases and deaths (2). Currently, two nucleoside analogs, remdesivir (RDV) and molnupiravir (MPV), both targeting viral RNA-dependent RNA polymerase, and one 3C-like (3CL) protease inhibitor, nirmatrelvir, have been developed for therapeutic treatment (3). Remdesivir (also named GS-5734), the first anti-SARS-CoV-2 drug approved by U.S. Food and Drug Administration (FDA), shows broad-spectrum antiviral activity against RNA viruses, including Ebola virus, Marburg virus, and Middle East respiratory syndrome coronavirus (MERS-CoV) (4). In contrast to remdesivir, which is given intravenously, the other two drugs, molnupiravir and nirmatrelvir, are administered orally (3). Nirmatrelvir has been approved by the FDA for the treatment of COVID-19 in adult patients at high risk for severe COVID-19, whereas molnupiravir has been granted emergency use authorization (5, 6). However, the wide clinical availability of these antivirals has led to increasing concerns about the emergence of drug-resistant viruses (7–9). Therefore, developing antivirals with alternative modes of action or synergistic drugs suitable for combination therapies poses a significant challenge in preparing for future pandemics.

SARS-CoV-2, a member of the *Betacoronavirus* genus, possesses a single-stranded positive-sense RNA genome of approximately 30 kb. RNA-dependent RNA replication results in the generation of 10 subgenomic RNAs, along with the full-length genomic RNA, ultimately transcribed to produce at least 29 proteins (10). The surface glycoprotein spike (S) of SARS-CoV-2 exists as trimers and consists of two subunits: the N-terminal S1 and the membrane-anchored C-terminal S2. Following proteolytic cleavage by furin at a polybasic cleavage site (P681–R685 or P**R**RA**R**↓) in SARS-CoV-2-infected producer cells, these two domains are non-covalently linked (11, 12). The S1 subunit contains a receptor-binding domain (RBD) responsible for attaching to the host cell surface by interacting with the cellular receptor, angiotensin-converting enzyme II (ACE2). S2, containing the fusion peptide and two heptad repeat regions (HR1 and HR2), plays a crucial role in membrane fusion during the entry step (13). The fusogenic property of S2 is activated by cleavage at a second proteolysis site (K811–R815 or KPS**KR**↓), named S2’, in human airway cells (12). This cleavage activation process is facilitated by a trypsin-like serine protease, transmembrane serine protease 2 (TMPRSS2), which promotes fusion between the plasma membrane and the virus envelope, ultimately leading to the release of viral genomic RNA into the cytoplasm. Alternatively, in a TMPRSS2-independent manner, the full-length S protein is cleaved at two novel cleavage sites by a cysteine protease, cathepsin L, for fusion with endo-lysosomal membranes in acidic conditions (14). These cleavage sites are located in flexible loop regions within the S1 subunit, one at amino acid 259 (cleavage site 1) and the other at amino acid 636 (cleavage site 2) near the S1/S2 cleavage site. Notably, it has been suggested that proteolysis at cleavage site 2 by cathepsin L is more crucial for membrane fusion (12, 14). In line with these findings, the involvement of cathepsin L in SARS-CoV-2 entry has been demonstrated through mRNA silencing or inhibitory small molecules, such as SID26681509 and E64d (15). Intriguingly, the analysis of protein-ligand cocrystal structures has proposed that cathepsin L inhibitors with hydrophobic moieties at the P1 site can also be accommodated in the S1 pocket of SARS-CoV-2 3CL protease, emphasizing the feasibility for developing dual inhibitors as SARS-CoV-2 antivirals (16).

In addition to the antiviral strategy directly targeting the catalytic function of cathepsin L in the S cleavage process, there is a suggestion that controlling pH-dependent endocytosis could be an attractive approach to efficiently prevent the entry of SARS-CoV-2 and other coronaviruses (17, 18). For instance, chlorpromazine, an inhibitor of clathrin-mediated endocytosis, has demonstrated the ability to block the internalization of ACE2-bound SARS-CoV particles (19). Moreover, the binding of small molecules, such as ouabain and bufalin, to Na^+^/K^+^-ATPase suppresses the entry of MERS-CoV and mouse hepatitis virus (20). More recently, it was suggested that derivatives of diphenylurea contributed to inefficient endocytosis of SARS-CoV-2 by a mode-of-action that stimulates the uptake of Cl^-^ by the cell, thereby disrupting intracellular homeostasis (21).

Based on the scientific understanding of the SARS-CoV-2 endocytic machinery and the published chemical structures of antiviral molecules, we conducted a screening of 1,459 FDA-approved compounds against SARS-CoV-2. Scoring for antiviral selectivity revealed that diphenoxylate most efficiently suppressed SARS-CoV-2 infection at non-toxic concentrations. Importantly, we demonstrated that the antiviral activity was induced by interfering with the endocytosis of viral particles bound to the cell membrane and by deacidification of endo-lysosomal vesicles. The positive charge on the tertiary amine of the diphenylmethyl compound was found to be essential for the latter case. In summary, we propose that diphenoxylate possesses a core structure deserving to further chemical modifications, leading to the development of an antiviral agent capable of inhibiting SARS-CoV-2 entry. Such an inhibitor could be employed as a monotherapy or in combination with other antiviral agents that operate through mechanisms distinct from existing anti-SARS-CoV-2 drugs.

## MATERIALS AND METHODS

### Viruses and cells

Original wild-type SARS-CoV-2 (hCoV-19/Korea/KCDC06/2020) and its variants (hCoV-19/Korea/KDCA51463/2021, KDCA55905/2021, KDCA95637/2021, KDCA119861/2021, and KDCA447321/2021) were provided by the Korea Disease Control and Prevention Agency. Vero CCL-81 (simply Vero) and Vero E6 cells (American Type Culture Collection, Rockville, MD, USA) were cultured at 37°C in Dulbecco’s modified Eagle’s medium (HyClone, South Logan, UT, USA) supplemented with 10% fetal bovine serum (FBS; Atlas Biologicals, Fort Collins, CO, USA). The human lung epithelial cell line Calu-3 (ATCC) was maintained in Eagle’s minimum essential medium (EMEM; Corning, Manassas, VA, USA) supplemented with 5% FBS. All experiments involving infectious SARS-CoV-2 were conducted in the KRICT biosafety level 3 facility.

### Compounds

The chemical library, which includes 1,459 clinically approved compounds, was provided by the Korea Chemical Bank (KCB, Daejeon, Republic of Korea). For the dose-response studies, powdered forms of meclizine, flunarizine, and manidipine with a purity greater than 95% were obtained from KCB. Control compounds, RDV (purity, 99.74%) and MPV (also known as EIDD-2801; purity, 99.94%), were purchased from MedChem Express (Princeton, NJ, USA). Nafamostat mesylate (purity, 98%) was obtained from Angene Chemical (Nanjing, China). Difenoxin was synthesized in-house through the saponification of diphenoxylate using NaOH. Briefly, diphenoxylate (31 mg, 0.07 mmol) was mixed with NaOH (26 mg, 0.67 mmol) in a solution of 0.4 mL methanol and 0.13 mL H_2_O and stirred at 60°C for 4 h. The reaction mixture was cooled and adjusted to pH 4.0 with 10% aqueous HCl. The resulting precipitate was collected by filtration, washed with H_2_O, and recrystallized in ethanol to yield difenoxin as a white powder (25 mg; purity, 99%). The identity of difenoxin was characterized by ^1^H NMR (Fig. S1).

### Screening of antiviral compounds

Overnight-cultured Vero cells in 96-well plates (2 × 10^4^ cells per well) were treated with 20 µM of each compound from the chemical library and simultaneously infected with SARS-CoV-2 at a multiplicity of infection (MOI) of 0.001. At 48 h post-infection, the cells were fixed and permeabilized for immunofluorescence assays using a mouse anti-S antibody (GeneTex, Irvine, CA, USA) and an Alex Fluor 488-conjugated goat anti-mouse IgG (Invitrogen, Carlsbad, CA) (22). The number of nuclei was measured by counterstaining with 4’,6-diamidino-2-phenylindole (DAPI; Invitrogen). The number of S-positive cells and nuclei detected in four separate areas per well was quantified in duplicate using the Operetta High Content Screening System (Perkin Elmer, Waltham, MA, USA) and the built-in Harmony software.

### Estimation of antiviral activity and cytotoxicity

Following the method described earlier, Vero cells in 96 wells were treated with threefold serial dilutions of each compound, ranging from 100 µM to 5 nM, both with and without SARS-CoV-2 infection at 0.01 MOI. The concentration of each compound that reduced the number of S-positive cells by 50% compared to dimethyl sulfoxide (DMSO)-treated cells was defined as the 50% effective concentration (EC_50_) (22). Concurrently, the viability of mock-infected cells was assessed using a 3-(4,5-dimethylthiazol-2-yl)−2,5-diphenyltetrazolium bromide (MTT) assay to determine the 50% cytotoxic concentration (CC_50_). The selectivity index (SI) is calculated as the ratio of CC_50_ to EC_50_.

### Detection of viral proteins and RNA

Immunoblot analysis was performed to detect viral proteins following the procedures outlined in our previous report, with some modifications (22). Briefly, Vero cells and Calu-3 cells cultured in six-well plates (2 × 10^5^ and 1 × 10^6^ cells per well, respectively) were infected with SARS-CoV-2 at an MOI of 0.001 for Vero cells or 0.1 for Calu-3 cells, at 37°C for 1 h. After the removal of the unabsorbed virus, the cells were treated with increasing concentrations of diphenoxylate, using remdesivir as a contro1. After 1 day for Vero cells or 2 days for Calu-3 cells, Viral S and nucleocapsid (N) proteins were detected using appropriate primary antibodies (GeneTex and Sino Biological, Beijing, China, respectively), followed by a horseradish peroxidase (HRP)-conjugated goat anti-mouse IgG antibody (Invitrogen). Changes in viral RNA copy numbers in the culture supernatant of cells or organoids were determined by one-step reverse-transcription quantitative PCR (RT-qPCR) using *N*-specific primers, as reported previously (23). Lung viral RNA was quantified according to our previous protocol with some modifications (24). From the total cDNA, the SARS-CoV-2 *S* gene was amplified with specific primers (5′-CAATGGTTTAACAGGCACAGG-3′ and 5′-CTCAAGTGTCTGTGGATCACG-3′), and its expression was normalized to the level of mouse β-actin mRNA using its corresponding primers (5′-CATTGCTGACAGGATGCAGAAGG-3′ and 5′-CCTCACTGTCCACCTTCCAGCA-3′).

### Time-of-addition experiment

Vero cells were cultured overnight in 48-well plates (5 × 10^5^ cells per well). They were treated with diphenoxylate (20 µM) for 2 h and then infected with SARS-CoV-2 (MOI of 1) for an additional 2 h (25). In parallel, cells were infected with SARS-CoV-2 for 2 h in the presence of diphenoxylate, either after a 30 min preincubation with the virus and the compound at room temperature, or without any preincubation. In a separate set of experiments, the compound was added to SARS-CoV-2-infected cells for 2 h. After each treatment step, the cells were washed with phosphate buffered saline (PBS) to remove non-specifically bound virus and compound. Culture supernatants were collected at 24 h post-infection to quantify viral titers through a plaque assay conducted on Vero E6 cells (23).

### Confocal microscopy

For the fluorescence microscopic analysis of virus-infected cells, SARS-CoV-2 was highly purified to remove cellular debris, following our previously reported methods with some modifications (23, 26). To visualize the distribution of N, Vero cells (6 × 10^4^ cells per well in four-well chamber slides) were infected with SARS-CoV-2 at an MOI of 10 in the presence of DMSO or 10 µM diphenoxylate. Cells were fixed and permeabilized at 0.5, 2, or 4 h post-infection. They were then incubated with mouse anti-N antibody (Sino Biological) and rabbit anti-early endosomal antigen 1 (EEA1) antibody (Santa Cruz Biotechnology; Santa Cruz, CA, USA). The primary antibodies were detected using Alexa Fluor 488-conjugated goat anti-mouse IgG and Alexa Fluor 633-conjugated goat anti-rabbit IgG (Invitrogen), respectively. Nuclei were counterstained with Vectashield mounting medium containing DAPI (Vector Laboratories, Burlingame, CA).

To analyze the detachment of SARS-CoV-2 particles from the cell membrane, Vero cells were pre-treated with DMSO as a mock control or 30 µM diphenoxylate at 37°C for 1 h. They were infected with SARS-CoV-2 at an MOI of 10 at 4°C for 30 min in the presence of DMSO or diphenoxylate. After washing PBS, the cells were incubated with the same treatment at 37°C for 8 h. Subsequently, the samples were treated with 2 µg/mL tosyl phenylalanyl chloromethyl ketone (TPCK)-treated trypsin for 1 h at the same temperature. After fixing and permeabilization, the viral N protein was immuno-fluorescently labeled as described above, with nuclei counterstained using DAPI.

To monitor pH changes in endo-lysosomal vesicles, Vero cells (8 × 10^4^ cells per 35 mm dish) were treated with either DMSO or 10 µM diphenoxylate for 1 h at 37°C. After washing with PBS, they were stained with acridine orange (8 µg/mL) for an additional 30 min. With excitation at 488 nm, live cell images were scanned at two wide-band emission wavelength ranges: 493–560 nm (green) and 590–720 nm (red). All fluorescent images were obtained using a Zeiss LSM 700 confocal microscope and analyzed with ZEN software (Carl Zeiss, Thornwood, NY, USA).

### Isobologram analysis

Diphenoxylate and nafamostat were mixed at various ratios: 5:0 (300 µM diphenoxylate only), 4:1, 3:2, 2:3, 1:4, or 0:5 (300 nM nafamostat only). These mixtures were serially diluted threefold, eight times, in EMEM and used to treat SARS-CoV-2-infected Calu-3 cells (MOI of 0.01). On day 1, cells were immunostained with an anti-S-antibody and its fluorescently labeled secondary antibody to estimate the two fractional EC_50_s (FEC_50_s) for each drug combination, as well as their sum (ΣFEC_50_). The interaction was defined as synergistic when the ΣFEC_50_ value was less than 0.8 (27).

### Antiviral assay using human tonsil organoids

According to our previous report (23), human tonsil organoids composed of 6 × 10^4^ cells were infected with SARS-CoV-2 at an MOI of 0.1 for 1 h at 37°C and then re-embedded in Matrigel (Corning Inc., Corning, NY, USA) in 48-well plates. The organoids were cultured for 2 days in 300 µL of medium supplemented with diphenoxylate or remdesivir as a control. Antiviral efficacy was evaluated by quantitative RT-PCR of the culture supernatants with the viral *N*-specific primers. All experiments involving human tissue-derived organoids were initiated after approval from the institutional review board of Konkuk University Hospital (IRB no. KUH110073).

### Pharmacokinetic studies in mice

Male ICR mice (8 weeks old; Orient Bio, Seongnam, Republic of Korea) were treated intravenously with diphenoxylate (5 mg/kg). Blood samples were collected at intervals of 0.5, 1, 2, 4, 8, and 24 h after treatment, and plasma was obtained by centrifugation at 13,000 rpm for 10 min at 4°C to remove cellular debris. The plasma concentrations of both diphenoxylate and difenoxin (in the same sample) were analyzed using LC-MS/MS, employing an HPLC (Agilent 1260; Agilent, Cheshire, UK) coupled with a mass spectrometer (Agilent 6460; Agilent). Calibration curves were prepared by spiking mock plasma with highly purified analytes, specifically diphenoxylate at 98% purity and difenoxin at 99% purity.

### *In vivo* efficacy study

Female mice (6 weeks old), expressing human ACE2 (hACE2) under the control of the human cytokeratin 18 promoter (K18) [B6.Cg-Tg(K18-ACE2)2Prlmn/J], were purchased from the Jackson Laboratory (Bar Harbor, ME, USA). Four hours before intranasal infection with SARS-CoV-2 (3 × 10^3^ plaque-forming units in 50 µL PBS per mouse), the mice received intranasal treatment with diphenoxylate at doses of 0.5 and 5 mg/kg (*n* = 3). Molnupiravir (25 mg/kg) dissolved in 200 µL of 0.1% carboxymethyl cellulose (CMC) was administered orally as a control. The mock and virus-only groups were treated intranasally with an equal volume of 50 µL PBS, matching the volume and frequency used for the diphenoxylate groups. Four hours after the virus challenge, these compounds were administered at the same doses. Treatment with diphenoxylate (twice a day at 1 and 10 mg/kg/day intranasally) and molnupiravir (twice a day at 50 mg/kg/day orally) continued on the following day (28, 29). On day 2 post-infection, each compound was administered at the same doses following the same regimen. After 8 h, all mice were sacrificed. Lung samples were collected for total RNA preparation and quantitative RT-PCR using *S* gene-specific and mouse *β-actin* gene-specific primers. All experimental procedures were approved by the KRICT Institutional Animal Care and Use Committee (IACUC; approval nos. 2022–6D-01–03 and 2023–6D-11–03).

### Statistical analysis

The statistical significance of differences between the two groups was analyzed using GraphPad Prism 8 (GraphPad, San Diego, CA, USA). *P* values were calculated by ANOVA followed by Dunnett’s multiple comparison test. For *in vivo* survival data, log-rank statistic *P* values were determined using Kaplan-Meier estimation. For all comparisons, a *P* value of less than 0.05 was considered significant.

## RESULTS

### Identification of diphenoxylate as an anti-SARS-CoV-2 agent

**Fig 1:**
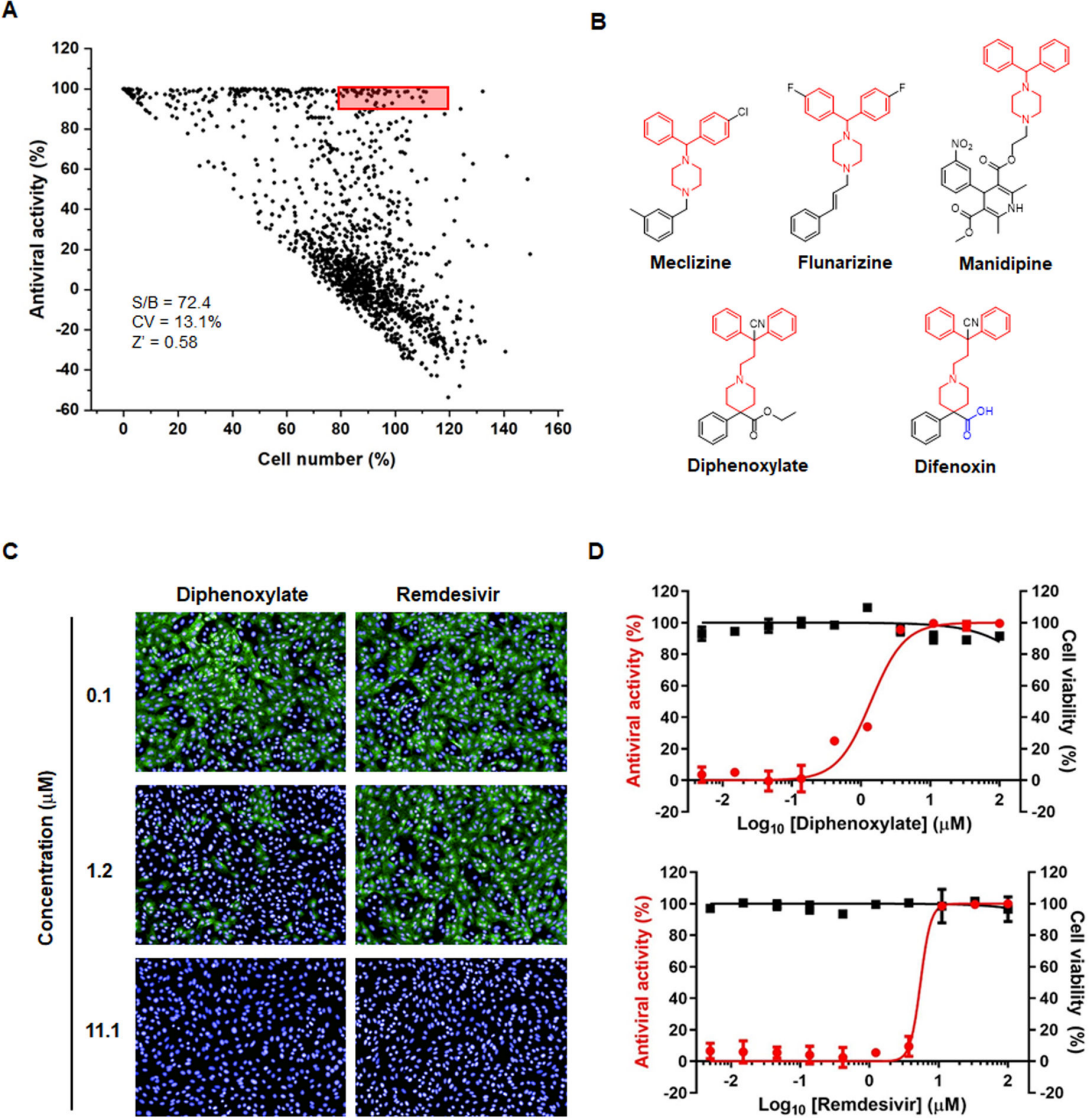
HTS of a chemical library identifies diphenoxylate as a SARS-CoV-2 inhibitor. (**A**) Cell culture-based screening of a chemical library comprising 1,459 clinically approved drugs to identify inhibitors of SARS-CoV-2. Vero cells infected with SARS-CoV-2 (MOI, 0.001) were treated with each compound (20 µM). On day 2 after treatment, the viral S protein was labeled with a mouse anti-S antibody and Alex Fluor 488-conjugated goat anti-mouse IgG, while cellular nuclei were counterstained with DAPI. Antiviral activity was determined by calculating the percentage inhibition of S protein expression relative to that in SARS-CoV-2-infected, DMSO-treated cells. The percentage of cells was determined by counting the number of DAPI-positive cells relative to the control. S/B, signal-to-background ratio. CV, coefficient of variation (%). Z’, average of the plate Z prime factors. Compounds with antiviral activity >90% and nuclei occupancy of 80%–120% are highlighted in the red box. (**B**) Chemical structures of the four hit compounds and difenoxin. The core chemical skeletons are colored red. The distinct chemical structure of the hydrolyzed product from diphenoxylate, difenoxin, is marked in blue. (**C** and **D**) Dose-dependent inhibition of SARS-CoV-2 infection by diphenoxylate. Vero cells infected with SARS-CoV-2 (MOI, 0.001) were treated with increasing concentrations of diphenoxylate or RDV as a control, ranging from 5 nM to 100 µM. (**C**) Representative images at concentrations of 0.1, 1.2, and 11.1 µM diphenoxylate (left) or RDV (right), where S protein is stained green and nuclei are stained blue. Original magnification, 20×. (**D**) Dose-response graph showing antiviral activity (red line) and cytotoxicity (black line) mediated by diphenoxylate (upper panel) and RDV (lower panel). Percent antiviral activity was determined as mentioned in (**A**). Percent cell viability was measured by treating Vero cells with each compound for 2 days using an MTT assay. All values are represented as mean ± SEM from three independent experiments.

To identify small molecule compounds exhibiting antiviral activity against SARS-CoV-2, we utilized a domestically isolated viral strain, hCoV-19/Korea/KCDC06/2020, closely related to the original Wuhan strain. We conducted a screening of 1,459 approved compounds (each at 20 µM) in SARS-CoV-2-infected Vero cell cultures. Immunofluorescence imaging with an anti-S antibody and nuclear counterstaining with DAPI revealed 45 hit compounds that reduced the S protein expression level by over 90%, while maintaining nuclei numbers at 80%–120% compared to the control (Fig. 1A). Molecular structure analysis showed that four hit compounds, including meclizine, flunarizine, manidipine, and diphenoxylate, shared a core structure comprising a diphenyl group bridged with a piperidine or piperazine ring (Fig. 1B). Particularly, the hit identification of previously reported compounds such as meclizine and flunarizine validated the reliability of our high-throughput screening (HTS) (30). Using highly purified, powdered compounds (purity, >95%), we treated SARS-CoV-2-infected Vero cells and mock-infected cells with threefold serial dilutions of each compound, ranging from 100 µM to 5 nM. Immunostaining with the anti-S antibody and cell viability tests with MTT were performed to estimate the EC_50_ and CC_50_ values, along with SIs. Results indicated that diphenoxylate exhibited antiviral activity at non-toxic concentrations (EC_50_, 1.4 µM; CC_50_, >100.0 µM; and SI, >71.4), surpassing the potency of other hits, including meclizine (EC_50_, 21.4 µM; CC_50_, 89.5 µM; and SI, 4.2), flunarizine (EC_50_, 12.9 µM; CC_50_, 30.6 µM; and SI, 2.4), and manidipine (EC_50_, 26.8 µM; CC_50_, 46.6 µM; and SI, 1.7; Table 1). Its antiviral efficacy (EC_50_, 1.4 µM) was more potent than that of remdesivir in Vero cells (EC_50_, 7.6 µM; CC_50_, >100.0 µM; and SI, >18.3; Table 1; Fig. 1C and D). Diphenoxylate also demonstrated reproducible efficacy against SARS-CoV-2 variants, such as Alpha, Beta, Gamma, Delta, and Omicron, with EC_50_ values ranging from 1.5 to 4.9 µM, making it up to 6.5 times more potent than remdesivir (Table 2). Therefore, diphenoxylate was selected for further evaluation of its antiviral efficacy in other infection systems and mode-of-action studies.

**TABLE 1:** Antiviral activity and cytotoxicity of the diphenyl piperidine and piperazine compounds against SARS-CoV-2

Compound	EC_50_ (μM)^*a*^	CC_50_ (μM)^*b*^	SI^*c*^	Mode of action
Meclizine	21.4 ± 3.0	89.5 ± 4.4	4.2	Antihistamine
Flunarizine	12.9 ± 5.7	30.6 ± 1.8	2.4	Antihistamine, calcium channel blocker
Manidipine	26.8 ± 1.1	46.6 ± 0.5	1.7	Calcium channel blocker
Diphenoxylate	1.4 ± 1.1	>100.0	>71.4	Anti-diarrheal, presynaptic opioid receptor agonist
Difenoxin	>100.0	>100.0	n.d.^*d*^	Metabolite of diphenoxylate
RDV	7.6 ± 1.2	>100.0	>18.3	Inhibition of viral RNA replication

^*a*^ Half maximal effective concentration necessary to reduce SARS-CoV-2 spike protein expression by 50%, calculated by nonlinear regression of dose-response inhibition from immunofluorescence images (*n* = 3).

^*b*^ Half maximal cytotoxic concentration necessary to reduce viability of Vero cells by 50%, calculated by nonlinear regression of dose-response inhibition from the MTT assay (*n* = 3).

^*c*^ Selectivity index, the ratio of CC_50_ to EC_50_.

^*d*^ Not determined.

**TABLE 2:** Antiviral activity of diphenoxylate against SARS-CoV-2 variants

Isolate	Clade	WHO label	EC_50_ (μM)^*a*^ (SI^*b*^)	
			Diphenoxylate	RDV
hCoV-19/Korea/ KDCA51463/2021	GR(B.1.1.7)	Alpha	1.9 ± 0.2 (>52.5)	7.0 ± 1.0 (>14.3)
hCoV-19/Korea/ KDCA55905/2021	GH(B.1.351)	Beta	4.9 ± 0.3 (>20.4)	12.2 ± 0.4 (>8.2)
hCoV-19/Korea/ KDCA95637/2021	GR(P.1)	Gamma	1.5 ± 0.1 (>68.0)	9.7 ± 0.7 (>10.3)
hCoV-19/Korea/ KDCA119861/2021	G(B.1.617.2)	Delta	2.8 ± 0.6 (>36.2)	4.2 ± 0.0 (>23.6)
hCoV-19/Korea/ KDCA447321/2021	GRA(B.1.1.529)	Omicron	3.2 ± 0.6 (>31.3)	1.6 ± 0.5 (>60.7)

^*a*^ Half maximal effective concentration required to reduce SARS-CoV-2 spike protein expression by 50% in Vero cells, calculated by nonlinear regression of dose-response inhibition from immunofluorescence images (*n* = 3).

^*b*^ Selectivity index, the ratio of CC_50_ to EC_50_.

### Efficient prevention of SARS-CoV-2 infection by pretreating cells with diphenoxylate

**Fig 2:**
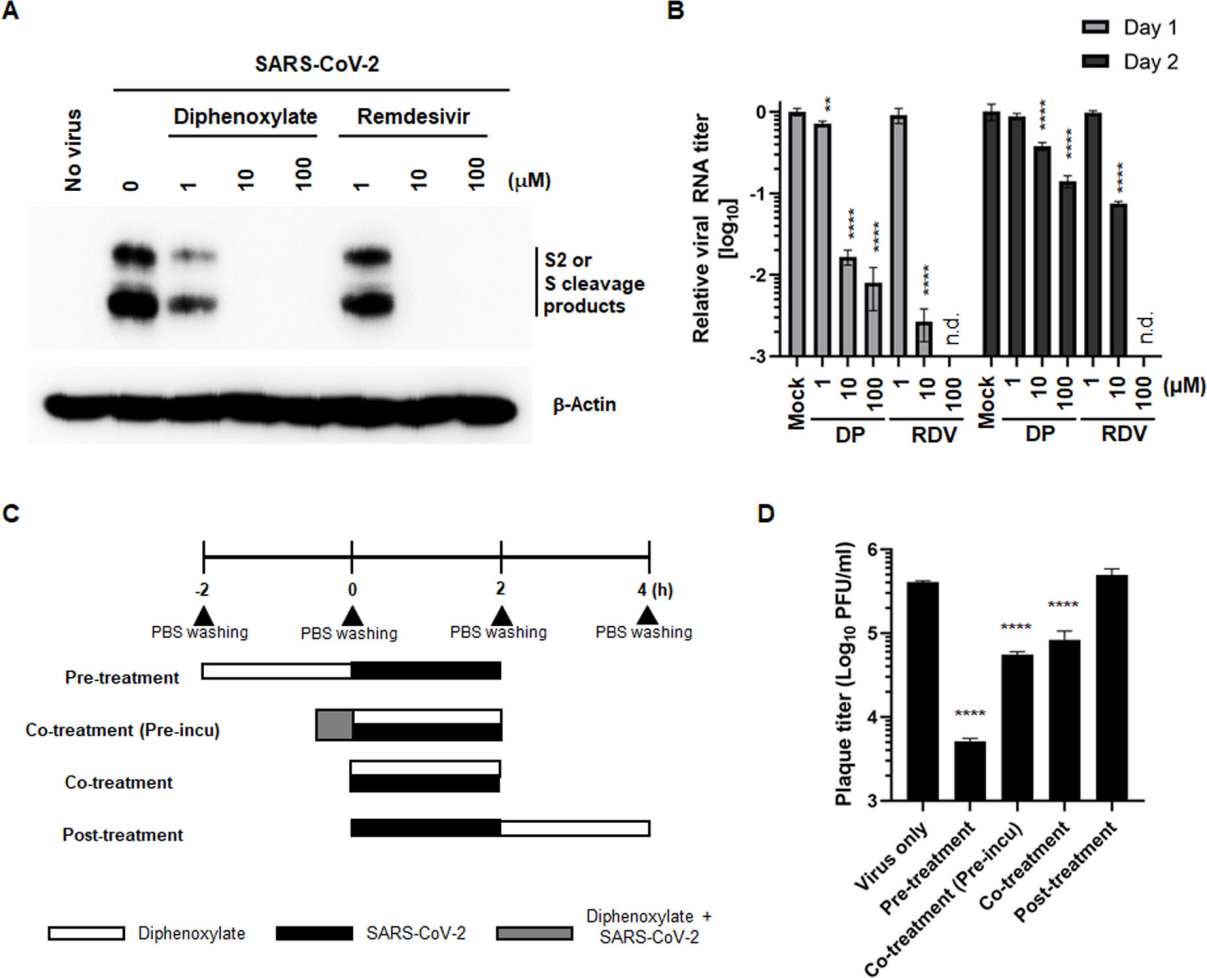
Diphenoxylate reduces viral protein and RNA levels as well as plaque formation of SARS-CoV-2. (**A**) Western blot analysis showing that diphenoxylate reduces the expression of the viral spike protein. Vero cells were mock-infected (no virus) or infected with SARS-CoV-2 (MOI, 0.001) and then treated with increasing concentrations (1, 10, and 100 µM) of diphenoxylate or RDV. The following day, cell lysates were harvested for immunoblotting with an anti-S antibody, followed by band detection with HRP-conjugated goat anti-mouse IgG. β-Actin was used as a loading control. Viral and cellular proteins are marked on the right side of the gels. (**B**) Quantitative RT-PCR showing reduced expression of viral RNA transcripts in the presence of diphenoxylate. Vero cell culture supernatants were prepared on days 1 and 2 post-infection, as mentioned in (**A**). Viral RNA encoding the *N* gene was amplified by one-step RT-PCR using specific primers. The amount of RNA in the presence of diphenoxylate is expressed relative to that in SARS-CoV-2-infected, mock-treated culture supernatants. n.d., not detected. (**C**) Schematic presentation illustrating the time-of-addition experiment. Vero cells were treated with 20 µM diphenoxylate (white box) for 2 h before infection with SARS-CoV-2 at an MOI of 1 (black box; pre-treatment). Cells were treated simultaneously with the compound and the virus (Co-treatment), or at 30 min after their preincubation [gray box; Co-treatment(Pre-incu)]. In another set of experiments, SARS-CoV-2-infected cells were treated with diphenoxylate at 2 h after virus infection (post-treatment). Unabsorbed compounds and viruses were removed by washing with PBS (black triangle). (**D**) Plaque titration. At 24 h after SARS-CoV-2 infection in (**C**), culture supernatants were harvested, and the viral titer was determined by infecting fresh Vero E6 cells with 10-fold serial dilutions of supernatant from each test sample. After 3 days, viral plaques were visualized using crystal violet. In (**B**) and (**D**), all values are expressed as the mean ± SEM from three independent experiments. Statistical significance was analyzed by two-way ANOVA with Dunnett’s multiple comparison test (**, *P* < 0.01; ****, *P* < 0.0001 vs the “mock” or “virus only” group).

Prior to conducting mode-of-action studies, we addressed concerns that diphenoxylate might be a false-positive hit, potentially causing a non-specific effect on fluorescence readouts during image-based HTS (31). To directly assess reductions in viral proteins and viral genome, we treated SARS-CoV-2-infected Vero cells with increasing concentrations of diphenoxylate or remdesivir as a control. Western blot analysis of cell lysates revealed that both compounds dose-dependently reduced the levels of S2 and additional S cleavage products (Fig. 2A). Concurrently, culture supernatants were collected to measure viral RNA copy numbers. Quantitative RT-PCR showed that both compounds efficiently suppressed the viral RNA copy numbers on days 1 and 2 compared to mock-treated, infected cells (Fig. 2B). These results confirmed that diphenoxylate is a genuine hit compound inhibiting SARS-CoV-2 infection.

Subsequently, a time-of-addition experiment was conducted to elucidate the stage of the viral life cycle targeted by diphenoxylate. In this experiment, diphenoxylate was introduced to cells under four distinct conditions: 2 h before viral infection (pre-treatment), during the 2 h viral infection period immediately after mixing with the virus (co-treatment) or with a 30 min preincubation at room temperature before co-treatment [co-treatment (pre-incu)], or 2 h after viral infection (post-treatment; Fig. 2C). At 24 h post-infection, culture supernatants were collected to quantify infectious viral titers using a plaque assay. The result revealed a substantial reduction in plaque numbers in the presence of diphenoxylate under pre-treatment, co-treatment (pre-incu), and co-treatment conditions but not under the post-treatment condition (Fig. 2D). The most pronounced antiviral activity, exemplified by an approximately two-log reduction in plaque numbers, was observed when diphenoxylate was added prior to SARS-CoV-2 infection. Collectively, these findings suggested that diphenoxylate could target the SARS-CoV-2 entry step by influencing a cellular molecule or environment rather than acting directly against incoming virus particles.

### Accumulation of SARS-CoV-2 on the cell surface and the deacidification of endo-lysosomal vesicles in the presence of diphenoxylate

**Fig 3:**
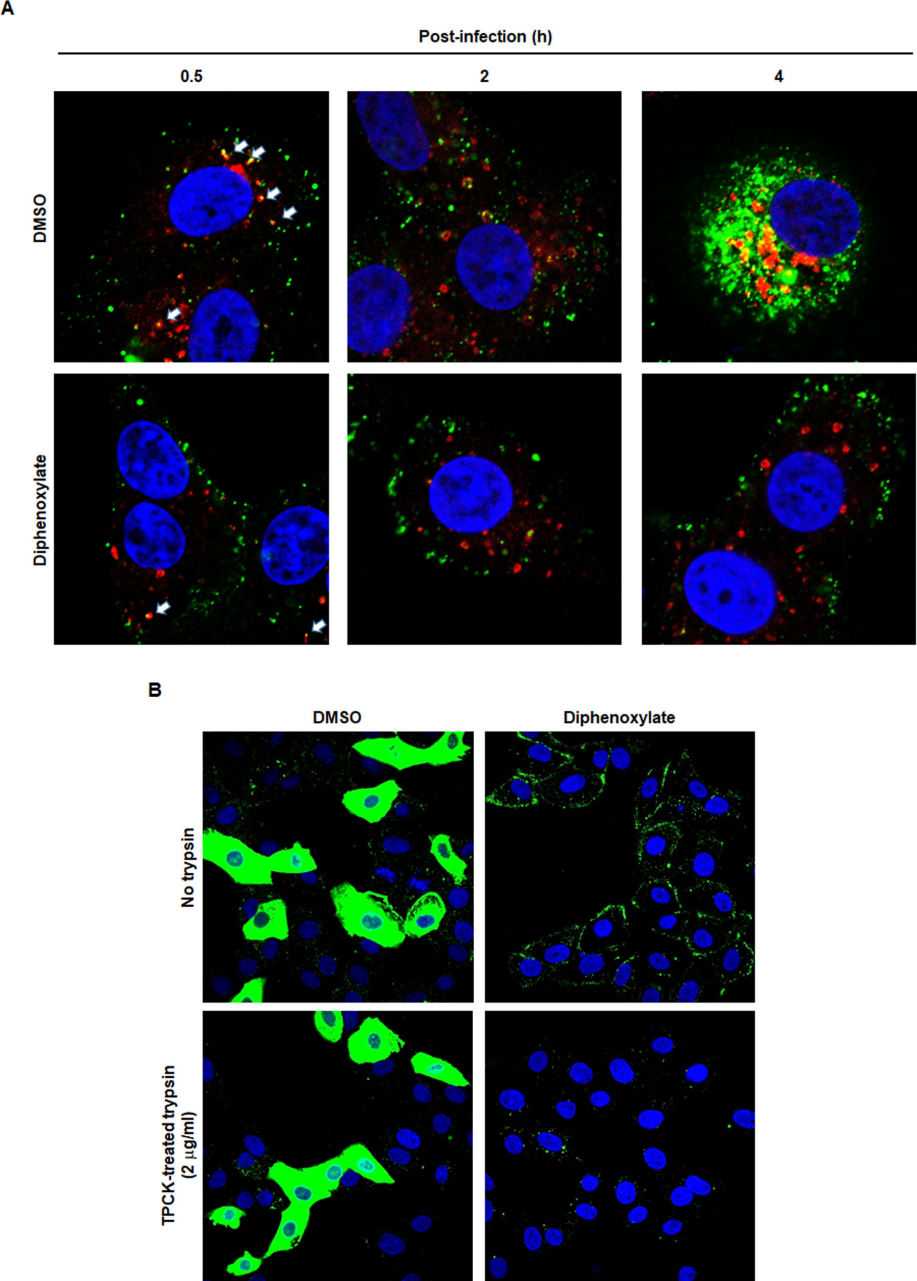
Diphenoxylate inhibits endocytic uptake of SARS-CoV-2. (**A**) Time-course study showing accumulation of viral N protein at the cell membrane. Vero cells were infected with highly purified SARS-CoV-2 at an MOI of 10 in the absence (upper) or presence of 10 µM diphenoxylate (lower). At 0.5 (left), 2 (middle), and 4 h (right) after infection, cells were fixed and permeabilized to detect viral N (green) and cellular EEA1 (red). Colocalization of N protein with EEA1 at 0.5 h is indicated by the white arrows. Nuclei were counterstained with DAPI (blue). Original magnification, 630×. (**B**) Detachment of membrane-bound SARS-CoV-2 particles following treatment with trypsin. Vero cells were treated with DMSO (left) or 30 µM diphenoxylate (right) at 37°C for 1 h prior to infection with SARS-CoV-2 at an MOI of 10 at 4°C for 30 min. After a PBS wash, the cells were treated with DMSO or diphenoxylate for an additional 8 h. Subsequently, they were incubated in the absence (upper) or presence of 2 µg/mL TPCK-treated trypsin (lower) for 1 h at the same temperature. Viral N protein was probed with its specific antibody and detected using Alexa Fluor 488-conjugated secondary antibody (green), while nuclei were counterstained with DAPI (blue). Original magnification, 630×.

To elucidate the specific sub-steps of SARS-CoV-2 entry affected by diphenoxylate, such as receptor binding, endocytosis, or fusion, we examined the distribution of the viral N protein in virus-infected Vero cells. Time-lapse fluorescence microscopy images showed that, in the absence of diphenoxylate, N efficiently colocalized with EEA1 at 0.5 h post-infection. Subsequently, the N protein was released into the cytoplasm between 2 and 4 h, leading to a robust increase in cytoplasmic N levels by 4 h (Fig. 3A, upper panels). In contrast, in the presence of diphenoxylate, only a small proportion of N colocalized with the early endosome marker at 0.5 h (Fig. 3A, lower left panel). It was predominantly detected on the cellular membrane, even at subsequent time points, 2 and 4 h post-infection, with marginal changes of its expression level (Fig. 3A, lower middle and right panels). At a later point, 8 h post-infection, an accumulation of SARS-CoV-2 on the membrane was also observed in the presence of diphenoxylate (Fig. 3B, upper panels). However, the SARS-CoV-2 N-derived fluorescence signals disappeared after trypsin treatment, confirming the localization of those viral particles on the outside of the cell membrane (Fig. 3B, lower panels). These findings underscore that diphenoxylate inhibits receptor-mediated endocytosis of SARS-CoV-2 without affecting the interaction between SARS-CoV-2 S and its receptor, ACE2.

**Fig 4:**
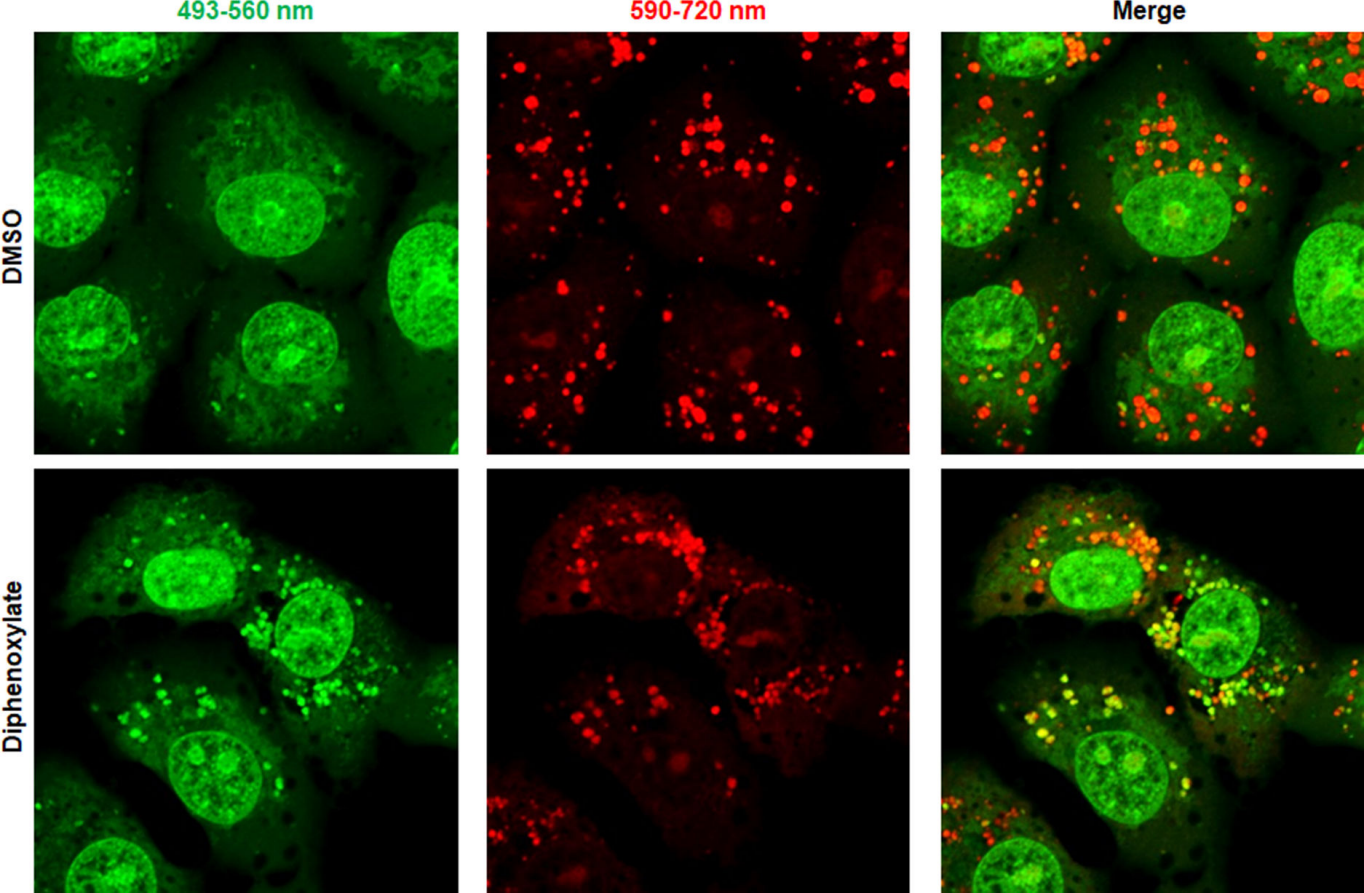
Deacidification of endo-lysosomal vesicles by diphenoxylate. Vero cells were treated with DMSO as a mock control (upper), or with 10 µM diphenoxylate (lower), at 37°C for 1 h. The live cells were labeled with acridine orange (8 µg/mL) for 30 min. Fluorescence images were acquired after excitation at 488 nm, at two different emission wavelengths: 493–560 nm (green; left) and 590–720 nm (red; middle). The images were merged (right) to distinguish acidic (red) and neutralized cytoplasmic vesicles (green or yellow) based on the red-to-green fluorescence intensity ratio. Original magnification, 630×.

Considering the structural characteristics of the four identified hit compounds presented in Fig. 1B, we hypothesized that protonation of the tertiary amine of diphenoxylate could hinder endo-lysosomal acidification if it permeates cells. To investigate this, we assessed its impact on the pH of cytoplasmic organelles using acridine orange, a membrane-permeable pH sensor. Acridine orange emits green fluorescence at 530 nm at low concentrations and red fluorescence at 680 nm by forming dimers at high concentrations (32). Given its pKa of approximately 9.65, protonated acridine orange, which does not permeate membranes, accumulates within acidic intracellular vesicles, resulting in red fluorescent puncta (33). Conversely, at higher pH levels, the unprotonated, membrane-permeable form of acridine orange fills vesicles with green monomeric dye at lower concentrations, visible as yellow or green merged spots. For comparative analysis, cells treated with either DMSO or diphenoxylate were incubated with acridine orange, and fluorescence images were acquired using two separate bandpass filters: one between 493 and 560 nm (representing neutral conditions, showing green fluorescence) and the other between 590 and 720 nm (representing acidic conditions, showing red fluorescence). As shown in Fig. 4, the merged image of DMSO-treated samples predominantly displayed cytoplasmic vesicles as red puncta (upper panels), while the diphenoxylate-treated sample exhibited an abundant population of vesicles appearing as yellow or green in the merged image (lower panels). This observation suggests that diphenoxylate neutralizes the acidic environment of endo-lysosomal organelles. To further investigate the significance of the positively charged diphenoxylate in deacidification, we synthesized its hydrolyzed form, difenoxin, as a negative control (Fig. 1B). Confocal microscopic analysis visualized that this zwitterinoic compound failed to deacidify endo-lysosomes of the same cell line, Vero, at the same concentration, 10 µM (Fig. S2). Consistent with this finding, the cell culture-based antiviral assay with difenoxin showed complete loss of antiviral activity against SARS-CoV-2 (Table 1). Collectively, these data strongly support the notion that diphenoxylate prevents SARS-CoV-2 entry by blocking receptor-mediated endocytosis and deacidifying endo-lysosomal vesicles, where the basic property conferred by the tertiary amine may play a critical role.

### Antiviral activity of diphenoxylate in human lung cells and tonsil organoids

**Fig 5:**
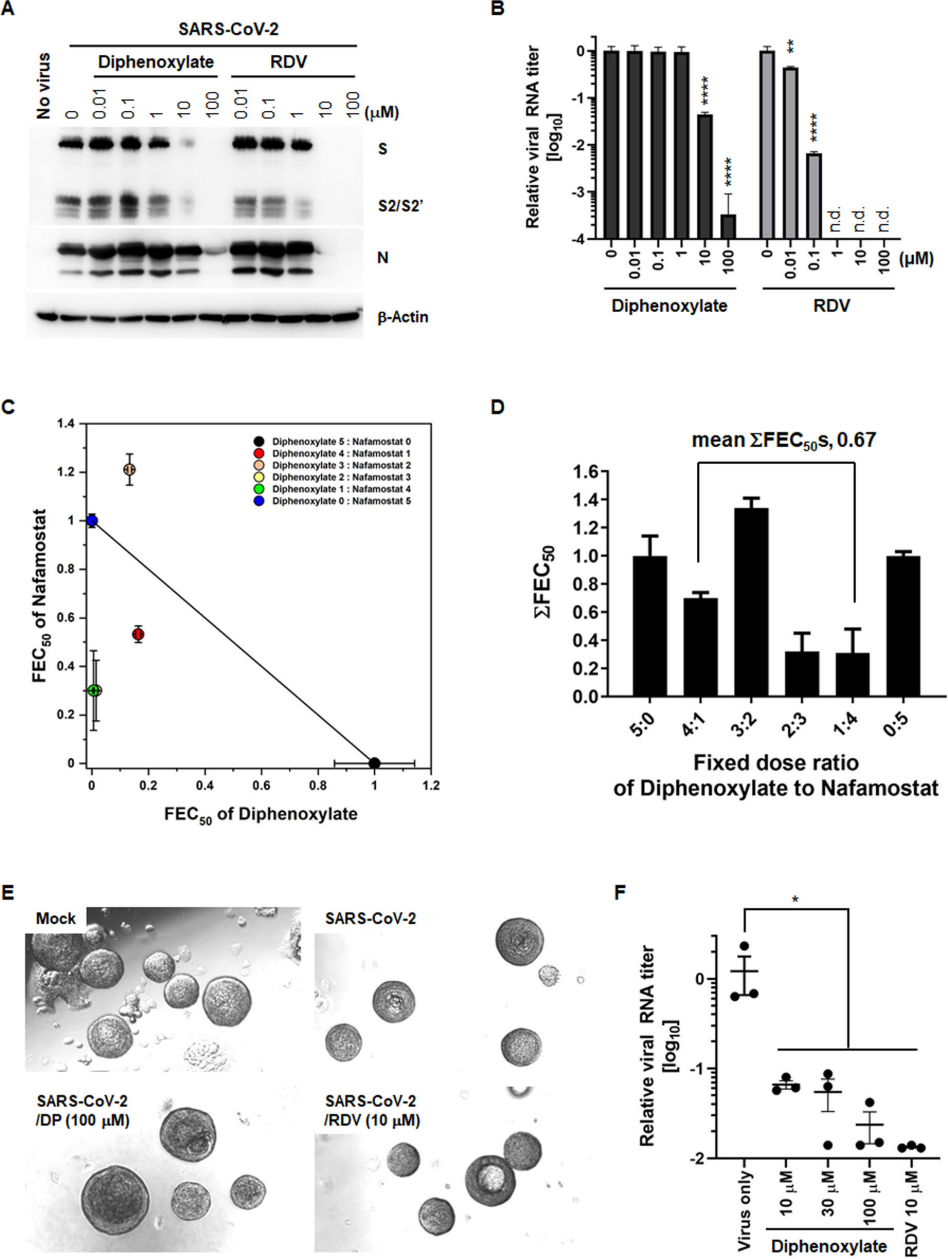
Anti-SARS-CoV-2 activity of diphenoxylate in human lung epithelial cells and tonsil organoids. (**A**) Western blot analysis showing antiviral activity of diphenoxylate. Calu-3 cells were mock-infected (no virus) or infected with SARS-CoV-2 (MOI, 0.1) and subsequently treated with increasing concentrations of diphenoxylate or RDV (0.001–100 µM). On day 2, cell lysates were harvested to detect both viral S and N proteins via western blot. β-Actin was used as a loading control. All probed proteins are marked on the right side of the gels. (**B**) Quantitative RT-PCR revealing reduced expression of viral RNA in the presence of diphenoxylate. Calu-3 cell culture supernatants, prepared as mentioned in (**A**), were used for amplification of viral RNA encoding the *N* gene by one-step RT-PCR. The relative amount of viral RNA in compound-treated cells vs mock-treated samples was determined. n.d., not detected. Values are expressed as the mean ± SEM from three independent experiments. Statistical significance was analyzed using two-way ANOVA with Dunnett’s multiple comparison test. **, *P* < 0.01; ****, *P* < 0.0001 compared to the mock-treated group. (**C**) Isobologram illustrating the synergistic activity of diphenoxylate and nafamostat against SARS-CoV-2. Mixtures of these two agents at various ratios were prepared as follows: 5:0, 300 µM diphenoxylate only; 4:1, 240 µM diphenoxylate and 60 nM nafamostat; 3:2, 180 µM diphenoxylate and 120 nM nafamostat; 2:3, 120 µM diphenoxylate and 180 nM nafamostat; 1:4, 60 µM diphenoxylate and 240 nM nafamostat; and 0:5, 300 nM nafamostat only. Each mixture was serially diluted threefold and added to virus-infected Calu-3 cells. The fractional EC_50_ (FEC_50_) indices were calculated for the different ratios. Data are expressed as the means ± SEM from three independent experiments. (**D**) The sums of both FEC_50_ values (ΣFEC_50_s) at fixed ratios. They are expressed as the mean ± SEM. Synergistic activity was defined as a mean ΣFIC_50_s < 0.8. (**E**) Representative images of tonsil organoids at day 2 post-infection with SARS-CoV-2. Organoids were mock-infected (upper left) or infected with SARS-CoV-2 for 1 h at an MOI of 0.1. The infected organoids were treated with DMSO (upper right), 100 µM diphenoxylate (DP; lower left), or 10 µM RDV (RBD; lower right). On day 2, live organoids were visualized under a conventional bright-field microscope. Original magnification, 100×. (**F**) Quantitative RT-PCR to detect viral RNA. Culture supernatants were harvested on day 2 and subjected to RT-PCR to detect the viral *N* gene. SARS-CoV-2-infected cells not exposed to diphenoxylate (virus only) were used as a positive control. Data represent the mean ± SEM from three independent experiments. Statistical significance was analyzed using one-way ANOVA with Dunnett’s multiple comparisons test compared to the virus-only control. *, *P* < 0.05.

As outlined in the introduction, SARS-CoV-2 utilizes two entry routes: a TMPRSS2-mediated early pathway and a pH-dependent, endosomal cathepsin L-mediated late pathway (34). This study indicates that diphenoxylate primarily affects the latter pathway in Vero cells, which express low levels of TMPRSS2 (35). To validate the reproducibility of the antiviral effect in another SARS-CoV-2-susceptible cell line with abundant TMPRSS2 expression, Calu-3 cells derived from human lung epithelium were infected with SARS-CoV-2 and treated with either diphenoxylate or remdesivir. Western blot analysis demonstrated that diphenoxylate suppressed the expression of viral proteins S and N dose dependently, albeit less potently than remdesivir (Fig. 5A). Correspondingly, viral RNA levels in culture supernatants decreased by one and three log orders of magnitude at 10 µM and 100 µM diphenoxylate, respectively, while remdesivir resulted in reductions exceeding four logs (Fig. 5B).

Furthermore, we explored the potential to enhance the antiviral activity of diphenoxylate in Calu-3 cells by combining it with a TMPRSS2 inhibitor, nafamostat. Preliminary studies identified the EC_50_ values of diphenoxylate and nafamostat to be 21.9 ± 2.1 µM and 3.5 ± 0.5 nM, respectively, with no cytotoxicity observed at the highest tested concentrations of 300 µM for diphenoxylate and 300 nM for naramostat (Fig. S3). These compounds were combined at various fixed dose ratios: 5:0 (300 µM diphenoxylate only), 4:1, 3:2, 2:3, 1:4, and 0:5 (300 nM nafamostat only). Each mixture was then serially diluted threefold at eight points for treatment of virus-infected Calu-3 cells. On day 1, FEC_50_ indices were calculated for each compound separately. Isobologram analysis showed synergistic effects for all combinations, except for the 3:2 ratio, which implied an additive effect (Fig. 5C). The summed FEC_50_ values for the mixtures were 0.7, 1.34, 0.32, and 0.31, respectively (Fig. 5D). Given that FEC_50_ values below 0.8 denote synergism, these data suggest that the antiviral effects of diphenoxylate and nafamostat are particularly improved when combined at concentrations of 120 µM and 180 nM (2:3 ratio) or 60 µM and 240 nM (1:4 ratio). The average of the summed FEC_50_s was 0.67, indicating a synergistic action that potentially blocks both TMPRSS2-mediated early and endosome-mediated late SARS-CoV-2 entry pathways.

We evaluated the antiviral activity of diphenoxylate using a human tonsil organoid model, previously validated as an *ex vivo* antiviral assay system for SARS-CoV-2 in our earlier study (23). The microscopic analysis confirmed that neither viral infection nor compound treatment impaired the 3D morphology of the tonsil epithelial organoids on day 2, thus ensuring organoid viability (Fig. 5E). Quantitative RT-PCR of virus-infected culture supernatants revealed reductions in viral RNA levels of between one and two logs in the presence of diphenoxylate and remdesivir (Fig. 5F). Taken together, these results suggest that diphenoxylate inhibits SARS-CoV-2 infection not only in TMPRSS2-expressing human lung cells but also in 3D cultures derived from tonsil epithelium organoids.

### Reduction of lung viral RNA titer in SARS-CoV-2-infected mice by diphenoxylate

**Fig 6:**
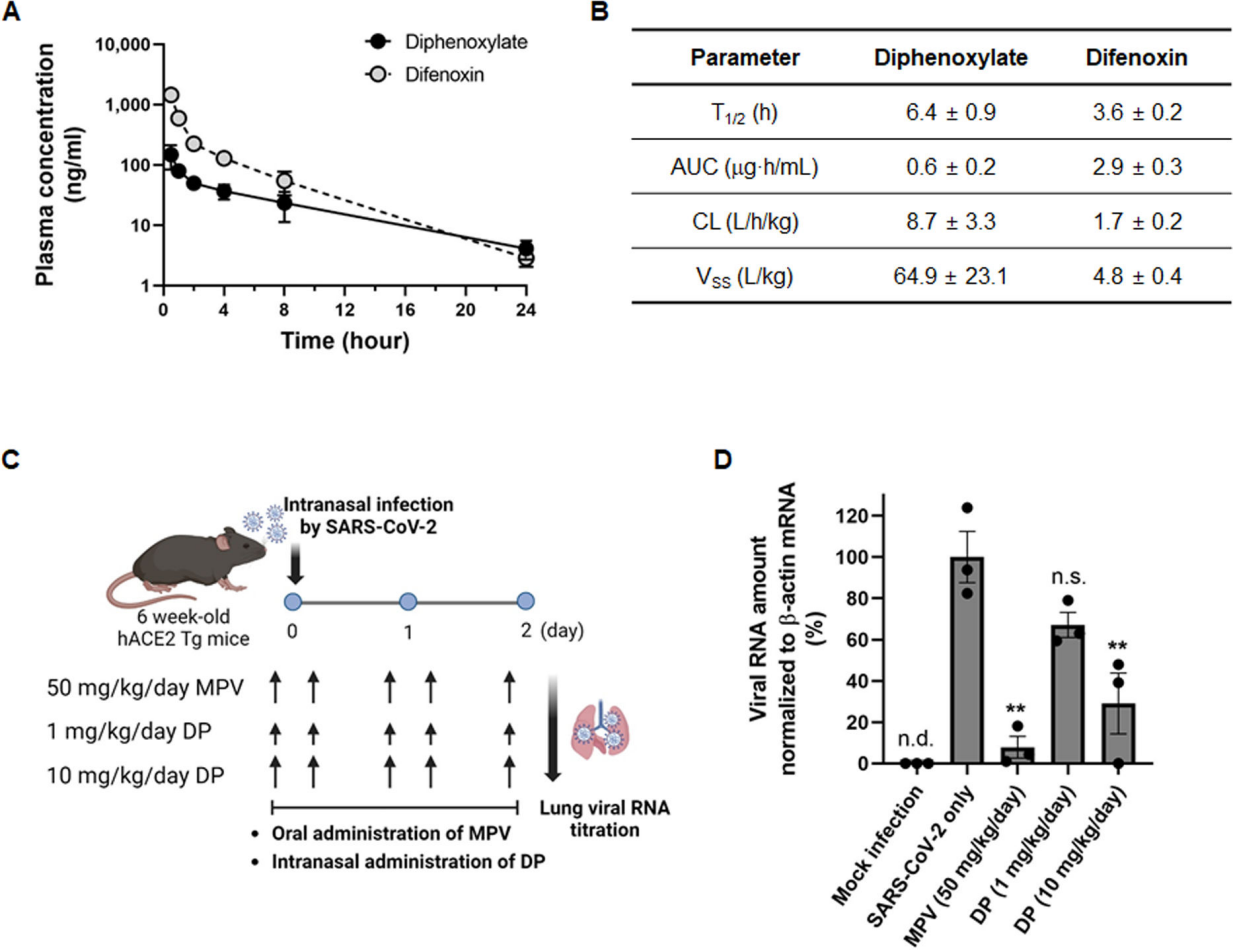
Pharmacokinetics and *in vivo* antiviral efficacy of diphenoxylate. (**A**) Plasma concentrations of diphenoxylate and its metabolite difenoxin. Diphenoxylate was administered to mice intravenously at a dose of 5 mg/kg. Concentrations of each compound at the indicated time points were measured using LC-MS/MS. (**B**) Quantitative analysis of the pharmacokinetic parameters of diphenoxylate and difenoxin. T_1/2_, terminal half-life in plasma; AUC, area under the concentration-time curve in plasma within 24 h after treatment; CL, total plasm clearance; V_SS_, volume of distribution under steady-state conditions. Values are expressed as the mean ± SEM from three mice. (**C** and **D**) Female hACE2-trangenic mice (6 weeks old; *n* = 3 per group) were mock-infected or intranasally challenged with SARS-CoV-2 (3 × 10^3^ plaque-forming units per mouse). Mice were then treated with PBS (SARS-CoV-2 only) or with diphenoxylate (DP; 1 and 10 mg/kg/day, BID) via the intranasal route. As a positive control, virus-infected mice were orally administered MPV at a dose of 50 mg/kg/day (BID). (**C**) Schematic illustration showing the timeline for compound treatment and lung sample preparation. (**D**) Quantitative RT-PCR to detect viral RNA in lungs. Lung samples, collected at day 2 post-infection, were analyzed by qRT-PCR to quantify the viral *S* gene, with normalization to β-actin mRNA levels. SARS-CoV-2-infected lung cells not exposed to antiviral compounds (SARS-CoV-2 only) served as a positive control. Data represent the mean ± SEM from three independent experiments. Two-way ANOVA with Dunnett’s multiple comparisons test was performed to analyze statistical significance compared with the SARS-CoV-2-only control. n.d., not detected; n.s., non-significant; **, *P* < 0.01.

Before assessing the *in vivo* therapeutic effects of diphenoxylate, we examined its pharmacokinetic stability in mice. Assuming hydrolysis of the ethyl ester group by serum or hepatic esterases, we measured the plasma concentrations of diphenoxylate and its metabolite difenoxin from samples collected at 0.5, 1, 2, 4, 8, and 24 h after intravenous administration. LC-MS/MS analysis revealed that approximately 90% of diphenoxylate was converted into the antivirally inactive difenoxin within 30 min after injection (Fig. 6A). This indicated poor serum stability and rapid metabolism of diphenoxylate [half-life (T_1/2_) of 6.4 h; area under the curve (AUC) of 0.6 µg·h/mL; clearance (CL) of 8.7 L/h/kg] to difenoxin (T_1/2_ of 6.4 h; AUC of 2.9 µg·h/mL; CL of 1.7 L/h/kg) in mice (Fig. 6B). Considering these pharmacokinetic results, we conducted an *in vivo* efficacy study in which SARS-CoV-2-infected hACE2 transgenic mice were administered diphenoxylate intranasally at doses of 1 and 10 mg/kg/day, twice daily (BID) for 2.5 days, while the control compound, molnupiravir, was administered orally at 50 mg/kg/day, BID for 2.5 days (36). To verify the antiviral potency of diphenoxylate *in vivo*, viral RNA was quantified from the lungs of the SARS-CoV-2-infected mice on day 2 post-infection (Fig. 6C). Quantitative RT-PCR analysis showed that viral RNA levels were significantly reduced following administration of molnupiravir (50 mg/kg/day) and the higher dose of diphenoxylate (10 mg/kg/day) but not at the lower dose (1 mg/kg/day; Fig. 6D). Collectively, the data suggest that although diphenoxylate is easily converted into an inactive metabolite, difenoxin, during systemic circulation, it can effectively reduce the lung viral RNA levels in SARS-CoV-2-infected mice when administered intranasally.

## DISCUSSION

In this study, HTS of a chemical library against SARS-CoV-2 in cell culture identified diphenoxylate as an antiviral agent that hinders viral entry into host cells. Originally prescribed in combination with the anticholinergic agent atropine for diarrhea treatment, diphenoxylate is known to become active after hydrolysis into difenoxin in humans, according to pharmacokinetic studies. However, our findings indicate that it is unmetabolized diphenoxylate, not difenoxin, that acts as the antiviral substance against SARS-CoV-2 infection (Table 1). The mechanism by which diphenoxylate acts as an antiviral agent appears to be distinctive from that of its hydrolyzed product used to relieve diarrhea. While difenoxin affects the peristaltic reflex by binding to mu- and delta-opiate receptors in the ileum of patients with chronic diarrhea, our mode-of-action studies manifest that diphenoxylate disrupts both virus internalization via endosomes (endocytosis) and the acidification of intracellular organelles (Fig. 3 and 4) (37).

Considering that the antiviral efficacy of diphenoxylate is significantly influenced by its overall positive charge (Table 1), the basic tertiary amine may play a major role in disrupting pH homeostasis around plasma membranes or intracellular vesicular membranes. Given concerns about potential non-specific electrolyte imbalances, it is necessary to delineate how its inhibitory mechanism differs from those of well-known antiviral tertiary amine compounds, such as chloroquine and hydroxychloroquine. These compounds have primarily been used to prevent malaria infection or to treat autoimmune diseases like lupus and rheumatoid arthritis. Early in the COVID-19 pandemic, they were recognized as potent anti-SARS-CoV-2 agents in cell cultures and were considered promising candidates for drug repurposing (38, 39). However, therapeutic benefits for SARS-CoV-2-infected patients have been highly controversial, with globally randomized clinical trials reporting that hydroxychloroquine has “little or no effect on hospitalized COVID-19, as indicated by overall mortality, initiation of ventilation, and duration of hospital stay” (40). Concerns have also been raised about the side effects of chloroquine, including its anti-inflammatory and immunomodulatory functions, oxidative/nitrosative stress activity, and genotoxicity (41). Although diphenoxylate partially shares the antiviral mechanism of chloroquine or hydroxychloroquine, particularly in deacidifying endo-lysosomal organelles, these compounds inhibit SARS-CoV-2 entry differently. Diphenoxylate targets the cell membrane penetration of SARS-CoV-2, unlike chloroquine and hydroxychloroquine, which do not affect viral internalization (38). Moreover, diphenoxylate does not affect endo-lysosomal vacuole size (Fig. 4), whereas the latter two compounds significantly increase the size of both early and late endosomes by trapping incoming viruses in these compartments (38). Consequently, we propose that diphenoxylate might block SARS-CoV-2 entry mainly by specifically affecting receptor-mediated endosome formation and, to a lesser extent, by perturbing the acidic pH of targeted endo-lysosomes. It remains unclear, however, whether the inhibitory actions on endocytosis and acidification are linked or operate independently. Currently, we hypothesize that there might be a key target molecule expressed on the cellular membrane that facilitates the acidification of newly formed endo-lysosomes, providing a driving force for receptor-mediated endocytosis of SARS-CoV-2. To explore this hypothesis further, we are attempting to identify the protein bound to diphenoxylate using diazirine photo-cross-linking and Cu(I)-catalyzed click chemistry (42).

In terms of chemical structure, it is noteworthy that among the hit compounds displaying structural similarity, diphenoxylate inhibited SARS-CoV-2 most potently and ensured a safety margin over a broader range of concentrations compared to other hits such as meclizine, flunarizine, and manidipine (Table 1). This suggests, as reiterated in the context of chloroquine and hydroxychloroquine, that although all of these diphenyl compounds contain a tertiary amine, the antiviral function may not be solely determined by a single factor related to the positive charge. The antiviral assay provided further insights, revealing that another tertiary amine compound, pridinol, displayed markedly reduced activity (EC_50_, approximately 96.4 µM; Fig. S4). Unlike diphenoxylate, pridinol has a hydroxyl group instead of a cyano group and a simple piperidine ring with no side chains. Thus, it appears that there are at least two additional elements contributing to the pronounced antiviral potential of diphenoxylate: the electron-withdrawing cyano group at the diphenyl-bridging carbon center and the ethyl carboxylate on the piperidine ring.

With regard to the antiviral potency of manidipine, a calcium channel blocker (Fig. 1B; Table 1), it has been reported to target the 3CL protease active site via virtual screening and to suppress the enzymatic activity *in vitro* (43). However, a more recently published report found that its inhibitory effect on the protease was not reproducible using the Flip-GFP and Protease-Glo luciferase assays, as well as the fluorescence resonance energy transfer (FRET)-based *in vitro* assay (44). Our HTS result revealed that manidipine is moderately active against SARS-CoV-2 infection into Vero cells, with an EC_50_ value of 26.8 µM (Table 1). Nevertheless, it has a narrow therapeutic window (SI, 1.7) due to inherent cytotoxicity (CC_50_, value of 46.6 µM). Based on structural similarity with diphenoxylate, we surmise that manidipine could potentially interfere with SARS-CoV-2 entry rather than inhibit 3CL protease activity. However, further validation studies with its less toxic derivatives are still needed.

We propose that diphenoxylate demonstrates significant antiviral selectivity (SI > 71.4; see Table 1). However, its clinical application as an anti-SARS-CoV-2 agent faces pharmacological limitations. These include poor metabolic stability and adverse effects characterized by central nervous system and respiratory depression in infants and children (45, 46). Our ongoing studies are focused on chemically modifying the ethyl carboxylate site to enhance metabolic stability while maintaining antiviral efficacy. Additionally, we are exploring the encapsidation of diphenoxylate within nano-sized particles for intranasal delivery. This approach aims to facilitate the controlled release of unmetabolized diphenoxylate near the lung epithelium, minimizing loss at the nasal mucosa. The data presented herein suggest that diphenoxylate can serve as a foundational skeleton for developing anti-SARS-CoV-2 agents, providing a rationale for further chemical modifications to discover alternative, clinically applicable antiviral drugs.

## SUPPLEMENTAL MATERIAL

Supplemental figures aac.00341-24-s0001.pdf Figures S1 to S4.

ASM does not own the copyrights to Supplemental Material that may be linked to, or accessed through, an article. The authors have granted ASM a non-exclusive, world-wide license to publish the Supplemental Material files. Please contact the corresponding author directly for reuse.
