# Supplementary material for: Inhibition of endocytic uptake of severe acute respiratory syndrome coronavirus 2 and endo-lysosomal acidification by diphenoxylate
Source: Antimicrob Agents Chemother. 2024 May 14;68(6):e00341-24. doi: 10.1128/aac.00341-24 (PMC11620506; doi:10.1128/aac.00341-24)
Supplement: Supplemental figures — Figures S1 to S4. [file aac.00341-24-s0001.pdf]

Supporting Information

Inhibition of endocytic uptake of severe acute respiratory syndrome coronavirus 2 and endo-lysosomal acidification by diphenoxylate

Jin Soo Shin,[1] Yejin Jang,[1] Dong-Su Kim,[1] Eunhye Jung,[1] Myoung Kyu Lee,[1] Byungil Kim,[1] Sunjoo Ahn,[2] Yeonju Shin,[1] Susan Jang,[1] Chang Soo Yun,[1] Jongman Yoo,[3] Young Chang Lim,[4] Soo Bong Han,[1,*] Meehyein Kim[1,*]

[1]Infectious Diseases Therapeutic Research Center, Korea Research Institute of Chemical Technology (KRICT), Daejeon 34114, Republic of Korea

[2]Therapeutics & Biotechnology Division, Korea Research Institute of Chemical Technology (KRICT), Daejeon 34114, Republic of Korea

[3]CHA Organoid Research Center, CHA University, Seongnam, Gyeonggi-do 13557, Republic of Korea

[4]Department of Otorhinolaryngology-Head and Neck Surgery, the Research Institute, Konkuk University School of Medicine, Seoul 05030, Republic of Korea

Jin Soo Shin, Yejin Jang, and Dong-Su Kim contributed equally to this work.

*To whom correspondence should be addressed:

Meehyein Kim, Ph.D.

Address: Infectious Diseases Therapeutic Research Center, Korea Research Institute of Chemical Technology, 141 Gajeongro, Yuseong, Daejeon 34114, Republic of Korea

E-mail: mkim@krict.re.kr

Telephone: +82-42-860-7540

Soo Bong Han, Ph.D.

Address: Infectious Diseases Therapeutic Research Center, Korea Research Institute of Chemical Technology, 141 Gajeongro, Yuseong, Daejeon 34114, Republic of Korea

E-mail: sbhan@krict.re.kr

Telephone: +82-42-860-7133

Contents

Figure S1. ^{1}H NMR spectroscopic data of difenoxin

Figure S2. No effect of difenoxin on endo-lysosomal acidic pH of Vero cells

Figure S3. Antiviral activity assay of diphenoxylate and nafamostat against SARS-CoV-2 in Calu-3 cells

Figure S4. Antiviral activity assay of pridinol against SARS-CoV-2 in Vero cells

Supplementary Data

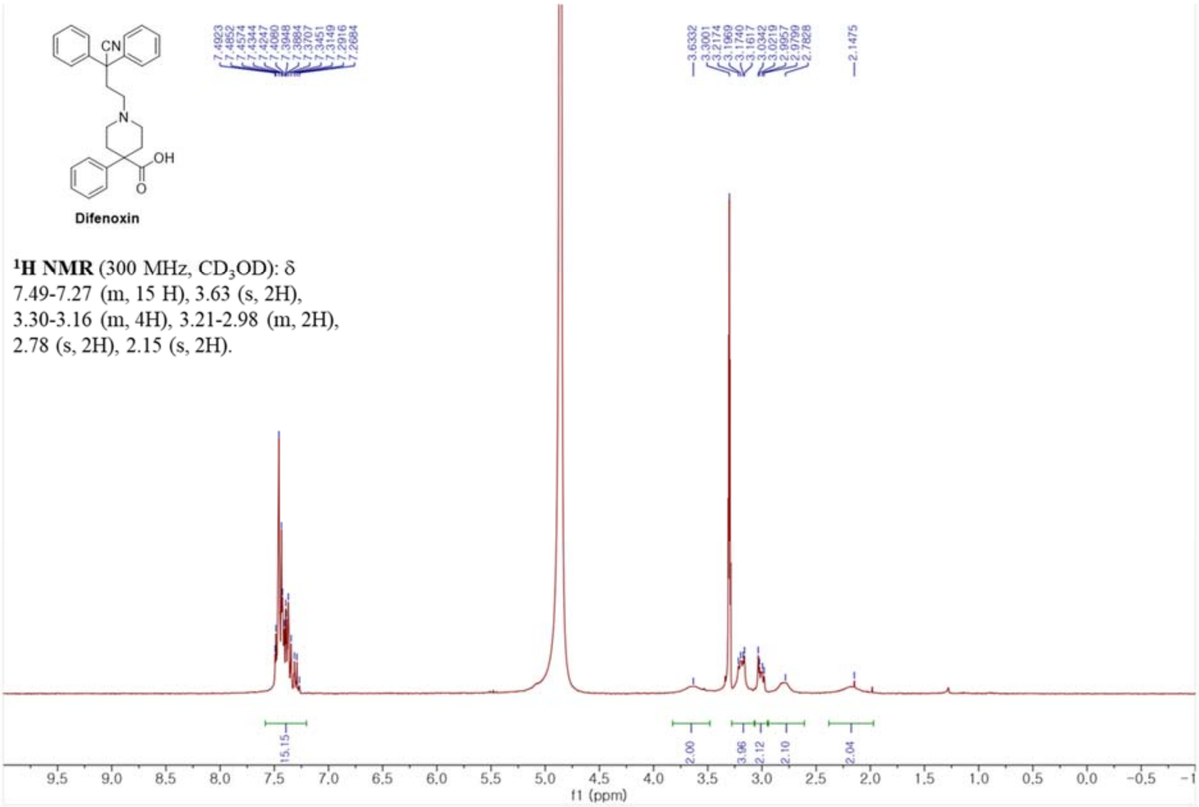

Figure S1. ^{1}H NMR spectroscopic data of difenoxin.

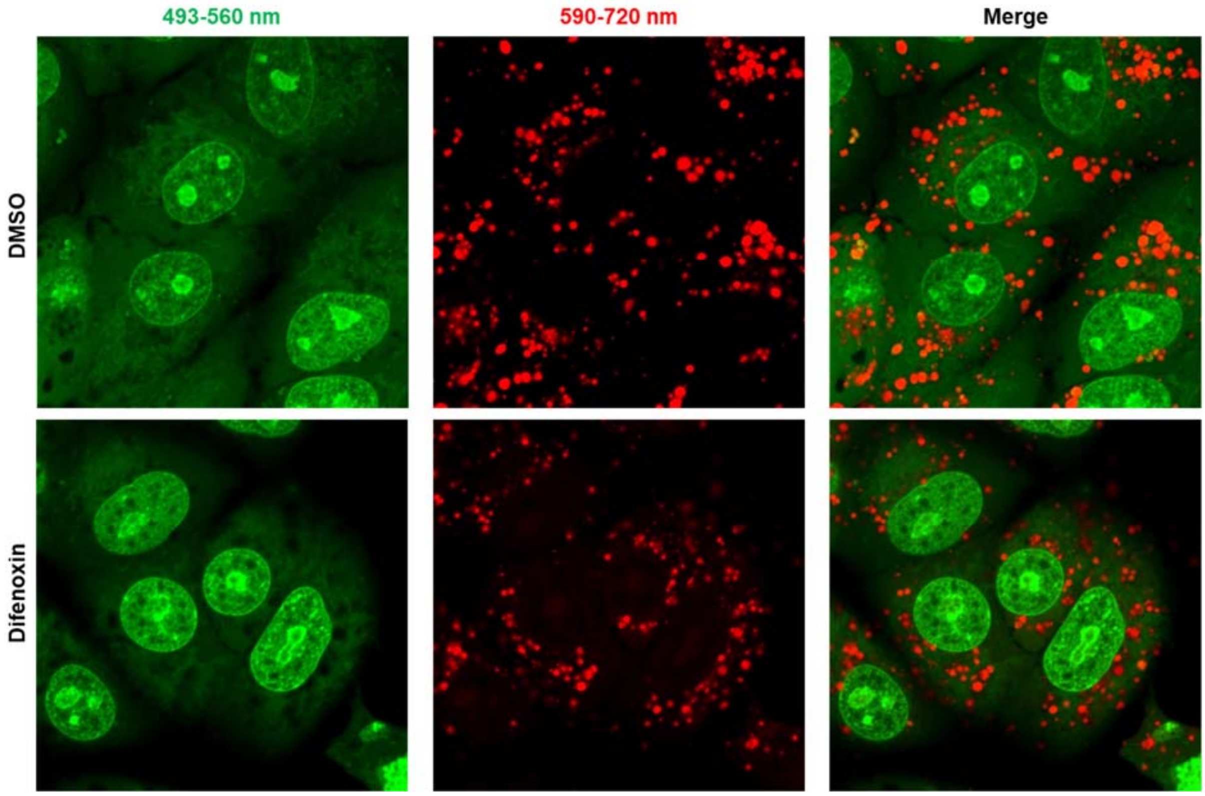

Figure S2. No effect of difenoxin on endo-lysosomal acidic pH of Vero cells. Vero cells were treated with DMSO as a mock control (upper), or with 10 µM difenoxin (lower), at 37℃ for 1 h. The live cells were then labeled with acridine orange (8 µg/mL) for 30 min. After excitation at 488 nm, fluorescence images were acquired at two different emission wavelengths: 493-560 nm (green; left) and 590-720 nm (red; middle). The images were merged (right) to distinguish acidic (red) and neutralized cytoplasmic vesicles (green or yellow) based on the red-to-green fluorescence intensity ratio. Original magnification, × 630.

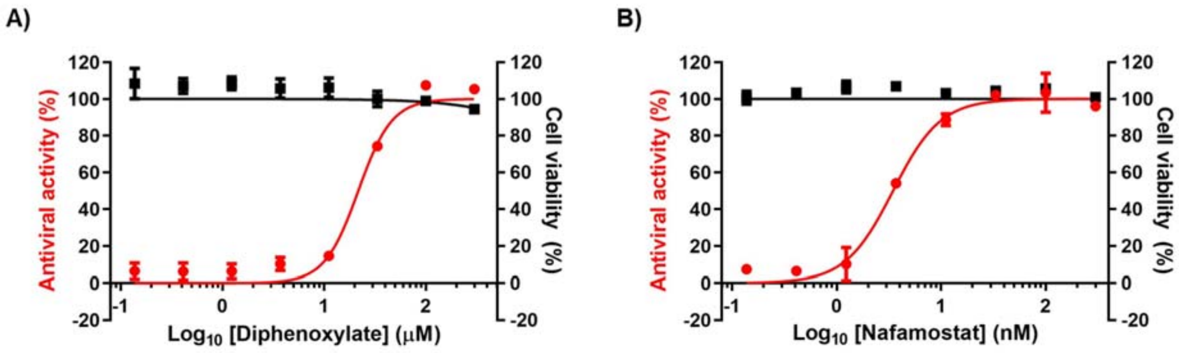

Figure S3. Antiviral activity assay of diphenoxylate and nafamostat against SARS-CoV-2 in Calu-3 cells. Calu-3 cells were either mock-infected or infected with SARS-CoV-2 (MOI, 0.1) and subsequently treated with increasing concentrations of diphenoxylate (A) or nafamostat (B). On Day 1 after treatment, the viral S protein was labeled with its specific antibody for an immunofluorescence assay, while mock-infected cells were incubated with MTT. Antiviral activity was determined by calculating the percentage inhibition of the S protein level relative to that in SARS-CoV-2-infected, DMSO-treated cells (red line). Cell viability was determined by measuring cell metabolic activity using MTT relative to naive cells (black line). All values are represented as the mean ± SEM from three independent experiments.

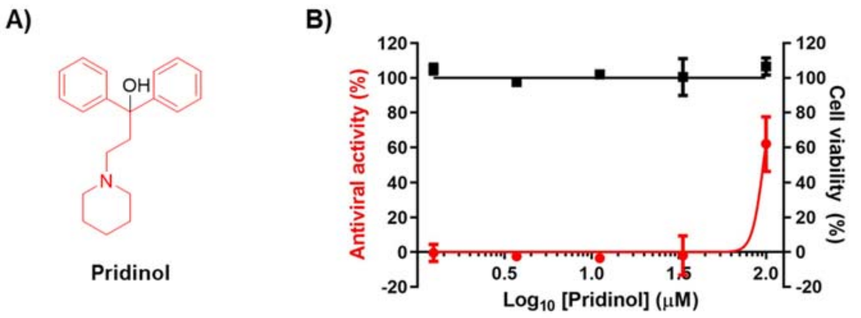

Figure S4. Antiviral activity assay of pridinol against SARS-CoV-2 in Vero cells. (A) Chemical structure of pridinol. The core skeleton, found in the hit compounds as shown in Figure 1B, is highlighted in red. (B) Antiviral activity and cytotoxicity of pridinol. Vero cells were mock-infected or infected with SARS-CoV-2 (MOI, 0.001) and then treated with increasing concentrations of pridinol. On Day 2 after treatment, the viral S protein was labeled with its specific antibody for an immunofluorescence assay, while mock-infected cells were incubated with MTT. Antiviral activity was determined by calculating the percentage inhibition of the S protein relative to that in SARS-CoV-2-infected, DMSO-treated cells (red line). Cell viability was measured by assessing cell metabolic activity using MTT relative to naive cells (black line). All values are represented as the mean ± SEM from three independent experiments.
